# Supplementary material for: A unifying Bayesian framework for merging X-ray diffraction data
Source: Nat Commun. 2022 Dec 15;13:7764. doi: 10.1038/s41467-022-35280-8 (PMC9755530; doi:10.1038/s41467-022-35280-8)
Supplement: Supplementary file 1 — Supplementary Information [file 41467_2022_35280_MOESM1_ESM.pdf]

Supplementary Information

Kevin M. Dalton[1], Jack B. Greisman[1], Doeke R. Hekstra[1,2]*.

[1]Department of Molecular & Cellular Biology, Harvard University, Cambridge, MA 02138

[2]John A. Paulson School of Engineering and Applied Sciences, Harvard University, Cambridge, MA 02138

*Corresponding author e-mail: doeke_hekstra@harvard.edu

Supplementary Note: Summary of conventional X-ray diffraction data processing

For reference, we summarize the conventional workflow for conversion of diffraction images to a non-redundant set of structure factor amplitudes—a process known as data reduction. This involves sequential application of a series of algorithms. First, during indexing, the orientation of the crystal lattice is determined, starting from a subset of strong reflections in each image, mapping each observed reflection to its Miller index ((h, k, l), abbreviated here as h). Next, in geometry refinement the mismatch between predicted and observed reflection centroids is minimized by optimizing estimates of detector position, beam center, crystal unit cell parameters, goniometer rotation axis, and other geometric parameters. In integration, the intensities of the reflections are then estimated, either by summing the pixel values in a region around the predicted spot centroid or by fitting a profile model to each reflection. In either case the region around a reflection can be used to estimate the background contribution from X rays resulting from other processes, such as inelastic scatter and scatter by sample mounts, bulk liquid, or air. This background is typically subtracted from the raw integrated intensity to yield an intensity estimate for each predicted reflection.

Next, the integrated intensities need to be converted into structure factor amplitudes. Traditionally this is done in three steps. In scaling, scale parameters are learned per image to account for beam intensity fluctuations, crystal disorder, radiation damage, sample absorption, and other effects which vary systematically throughout a diffraction experiment. The parameters are applied to each reflection to yield scaled intensities. At this point, the data still contain many redundant observations for each Miller index. In merging, equivalent observations are merged by weighted averaging. This step assumes that the errors in the reflection intensities are normally distributed. This works well if the errors in the reflection intensities are dominated by photon counting statistics or other random additive errors, but is sensitive to outliers. In French-Wilson scaling, the merged intensities are then "corrected" because they can be negative, resulting from errors in the estimated intensity of reflections and background, and inconsistent with the reflection intensities being proportional to squared structure factor amplitudes, and therefore positive.

In this last step, the Wilson distribution[1] serves as a prior probability distribution, or prior. This prior is combined with a statistical model of the true intensity given the observed merged intensity to yield a posterior probability distribution, or posterior, of the true merged intensity. This true intensity, the squared structure factor amplitude, is guaranteed to be positive. The Wilson distribution is parametrized by the mean intensity which is usually modeled as resolution-dependent.

Supplementary Figures and Tables

Merging as a Graphical Model

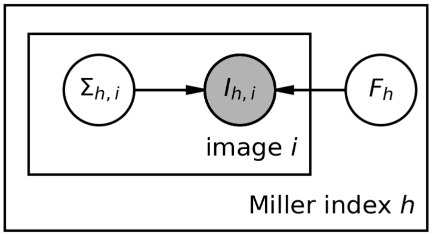

Observed Data

- $I_{h,i}$: Intensities
- $\sigma_{I_{h,i}}$: Uncertainties

Latent Variables

- $\Sigma_{h,i}$: Reflection scales
- F_h : Structure factor amplitudes

Supplementary Figure 1: **Merging Diffraction Data posed as a Graphical Model.**

Model Parameters

Scale function

θ : MLP kernels and biases

Structure factors

$\mu_{q_{F_h}}$: location

$\sigma_{q_{F_h}}$: scale

Model Parameterization

$$F_h \sim q_h$$
$$\Sigma_{h,i} \sim q_{\Sigma_{h,i}}$$
$$I_{h,i} \sim \text{Normal}\left(F_h^2 \Sigma_{h,i}, \sigma_{I_{h,i}}\right)$$

or

$$I_{h,i} \sim \text{StudentT}\left(d.f., F_h^2 \Sigma_{h,i}, \sigma_{I_{h,i}}\right)$$

Variational Distributions

$$q_{F_h} = \text{TruncNormal}(\mu_{q_{F_h}}, \sigma_{q_{F_h}}, 0, \infty)$$
$$\left[\mu_{\Sigma_{h,i}}, \sigma_{\Sigma_{h,i}}\right] = f_\theta\left(\mathbf{M_{h,i}}\right)$$
$$q_{\Sigma_{h,i}} = \text{Normal}\left(\mu_{\Sigma_{h,i}}, \sigma_{\Sigma_{h,i}}\right)$$

Priors

$$F_h \sim \text{Wilson}(h, \epsilon_h)$$
$$\Sigma_{h,i} \sim q_{\Sigma_{h,i}}$$

Supplementary Figure 2: **Summary of the model parameterization.**

```python
steps=10000
n_layers = 20
mc_samples = 3
p_centric  = tfd.HalfNormal(np.sqrt(multiplicity))
p_acentric = tfd.Weibull(2., np.sqrt(multiplicity))

#Construct variational distributions
loc_init   = tf.where(centric, p_centric.mean(), p_acentric.mean())
scale_init = tf.where(centric, p_centric.stddev(), p_acentric.stddev())
q = tfd.TruncatedNormal(
    loc = tf.Variable(loc_init),
    scale = tfp.util.TransformedVariable(scale_init, tfp.bijectors.Softplus()),
    low = tf.where(centric, 0., 1e-30),
    high = 1e30,
)

#Construct error model
likelihood = tfd.Normal(loc=intensities, scale=uncertainties)

#Construct scale function
n,d = metadata.shape
NN = tf.keras.models.Sequential()
NN.add(tf.keras.Input(d))
for i in range(n_layers):
    NN.add(tf.keras.layers.Dense(d, kernel_initializer='identity'))
NN.add(tf.keras.layers.Dense(2, kernel_initializer='identity'))

#Evaluate the elbo
def minus_elbo():
    z = q.sample(mc_samples)
    F = tf.gather(z, miller_id, axis=1)
    loc, scale = tf.unstack(NN(metadata), axis=1)
    Sigma = tfd.Normal(loc, scale).sample(mc_samples)
    log_likelihood = tf.reduce_sum(likelihood.log_prob(F * F * Sigma))
    log_p_z = tf.where(centric, p_centric.log_prob(z), p_acentric.log_prob(z))
    log_q_z = q.log_prob(z)
    kl_div = tf.reduce_sum(log_q_z - log_p_z)
    return -log_likelihood + kl_div

#Train the model
optimizer = tf.keras.optimizers.Adam()
for i in range(steps):
    optimizer.minimize(minus_elbo, [q.trainable_variables , NN.trainable_variables])

#Export the results
F,SigF = q.mean().numpy(), q.stddev().numpy()
```

Supplementary Figure 3: **Example implementation of the Careless model using TensorFlow Probability**. This example code is fully functional and available as a script on the Careless GitHub page.[2]

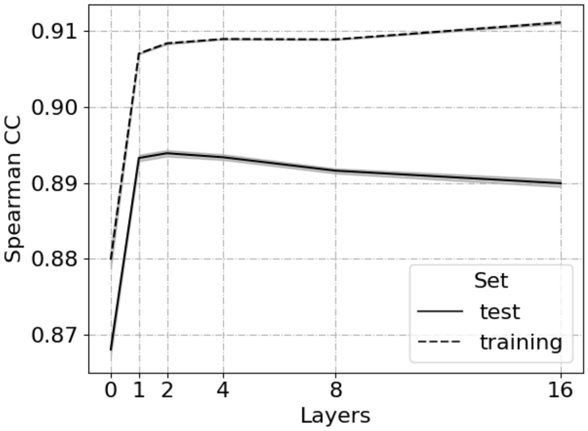

Supplementary Figure 4: **Selection of the number of image layers by 10-fold cross-validation of observed against predicted intensities for merging thermolysin serial crystallography.** Shaded region represents bootstrapped 95% confidence intervals of the mean.

(a)

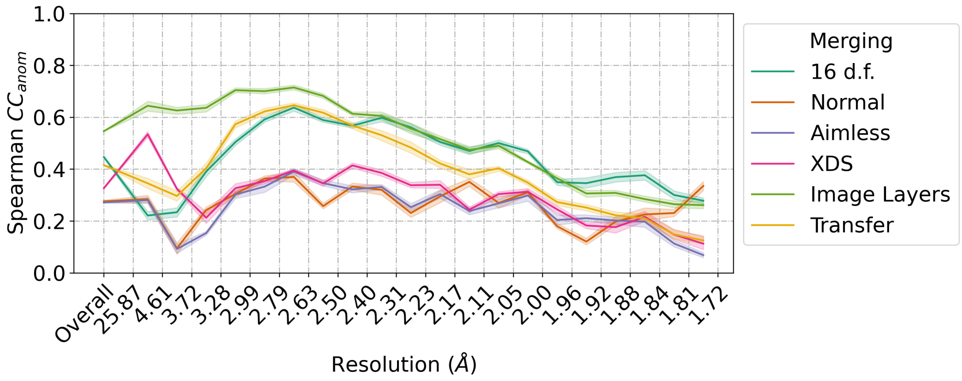

(b)

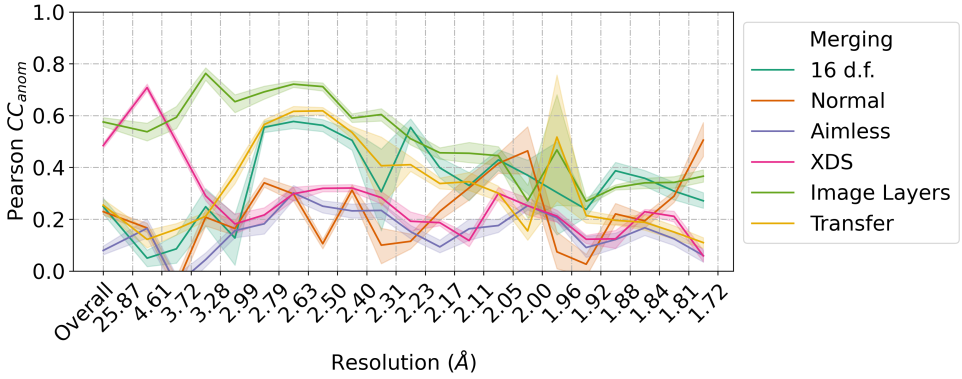

(c)

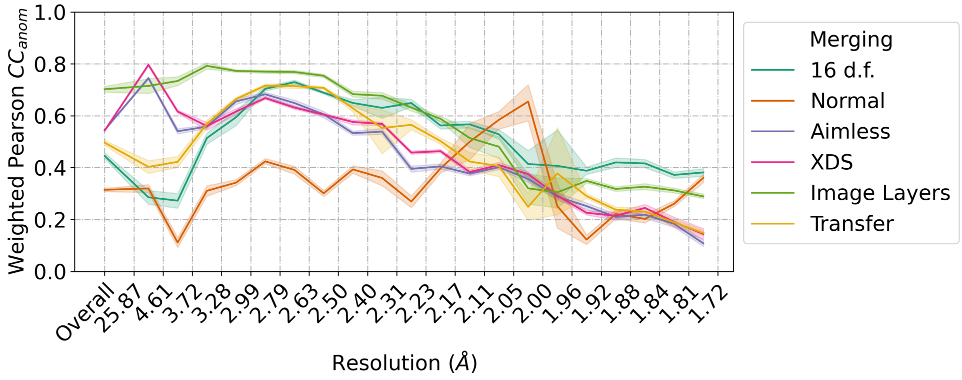

Supplementary Figure 5: **Anomalous correlation coefficients CC_{anom} by three statistical measures for various scenarios.** Lines: average values; semitransparent bands: 95% confidence intervals by the bootstrap method from 10 merging repeats with different half-dataset partitions. (a) The Spearman correlation coefficient, (b) Pearson correlation coefficient, and (c) inverse variance-weighted Pearson correlation coefficient, approximating the calculation of CC_{anom} in XDS.

(a) XDS

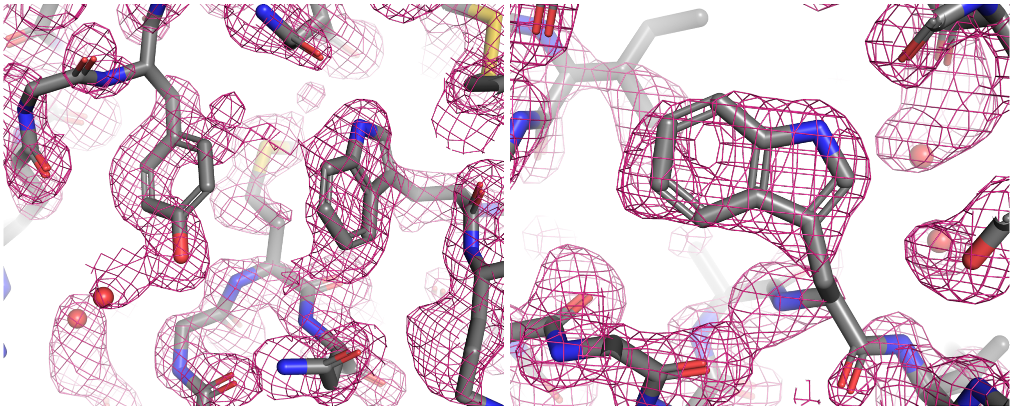

(b) Image Layers

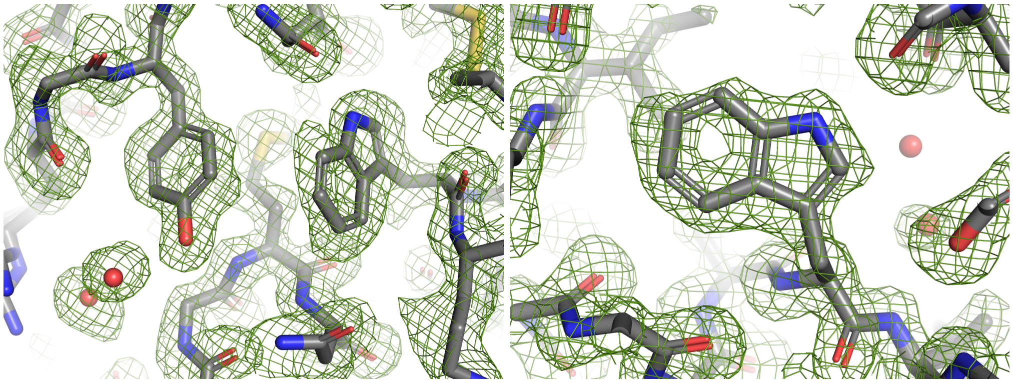

(c) Transfer

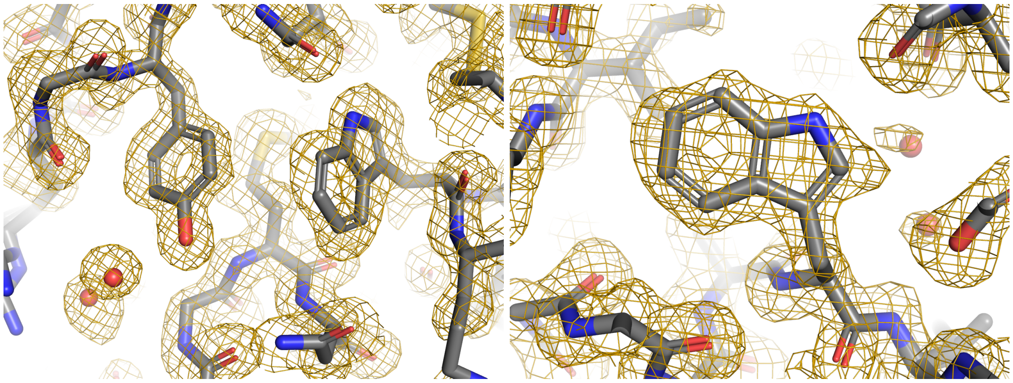

Supplementary Figure 6: **Density-modified experimental electron density maps.** Produced with PHENIX Autosol[3] using the sulfur substructure from a reference structure (PDBID: 7L84), contoured at 1.0 σ.

Degrees of Freedom	Figure of Merit	Bayes-CC ± S.D.	Sites
0.125	0.23	9.9 ± 12.3	10
0.25	0.19	15.8 ± 14.0	10
0.5	0.4	25.7 ± 14.4	11
1	0.21	13.3 ± 13.3	10
2	0.42	32.1 ± 13.7	10
4	0.43	31.6 ± 13.6	11
8	0.43	31.8 ± 13.6	10
16	0.42	33.8 ± 13.4	11
32	0.43	26.6 ± 14.6	11
64	0.43	32.6 ± 13.3	10
128	0.41	33.4 ± 13.3	10
256	0.42	28.3 ± 14.1	10
512	0.41	30.1 ± 13.9	10
1024	0.39	27.0 ± 14.2	11
∞	0.39	24.3 ± 14.5	11
Image layers	0.45	36.7 ± 11.7	10
Transfer	0.45	36.2 ± 11.7	10
Aimless	0.45	27.3 ± 14.4	11
XDS+AutoSol	0.44	27.0 ± 14.3	10
XDS+Careless (transfer)	0.44	29.6 ± 14.4	10
XDS+SHELXE	0.35	—	11

Supplementary Table 1: ***Ab initio* phasing results from AutoSol using thorough defaults and searching for 10 sulfur sites.** The Figure of Merit of phasing places an upper bound on the quality of an experimentally phased electron density map. The Bayes-CC is an effective estimate of the quality of an experimental map before density modification based on its skew. See[3] for further description of both metrics. The Image layers and Transfer entries describe are described on the careless-examples GitHub page. Briefly, Image layers uses 2 image layers and a 10-layer multilayer perceptron. The transfer protocol is a two step protocol wherein the data are first merged without anomalous signal. The scale model learned in the first run is used to merge the data keeping Friedel mates separate. For "XDS+AutoSol", data were indexed, integrated, scaled and merged in XDS, followed by phasing with AutoSol. For "XDS+Transfer", data were indexed, integrated, scaled and merged in XDS, followed by scaling and merging in Careless according to the "Transfer" protocol, and phased with AutoSol. For "XDS+SHELXE", data were indexed, integrated, scaled and merged in XDS, followed by phasing with SHELXC/D/E.

Site	Aimless	Careless (16 d.f.)	Careless (∞ d.f.)	Careless (image layers)	Careless (transfer)	XDS	XDS+ Careless (transfer)
Cys-6	14.91	11.97	10.45	14.48	15.86	12.72	15.35
Met-12	17.52	10.92	9.8	16.48	17.33	14.03	16.53
Cys-30	19.75	14.63	12.10	19.47	20.51	18.69	20.33
Cys-64	17.59	14.01	9.75	18.02	19.06	16.56	18.99
Cys-76	14.84	11.39	10.85	13.71	15.82	13.29	15.11
Cys-80	18.29	15.30	12.88	18.63	20.90	17.21	18.90
Cys-94	14.98	12.28	8.86	14.54	15.95	13.84	15.79
Met-105	19.89	14.84	11.67	20.30	21.48	17.92	20.16
Cys-115	19.38	16.13	11.41	18.92	20.29	17.47	19.50
Cys-127	14.62	10.64	10.16	14.50	15.35	11.78	14.29
Average	17.18	13.21	10.79	16.91	18.26	15.35	17.50

Supplementary Table 2: **Anomalous omit map peak heights from PHENIX refinement with isotropic B-factors and rigid body refinement.** The Careless merging options used here are described in detail on the careless-examples GitHub page. The XDS column contains the results from scaling and merging with XDS. For "XDS+Transfer", data were indexed, integrated, scaled and merged in XDS, followed by scaling and merging with Careless using the transfer learning protocol.

Wavelength (Å)	1.892
Resolution range	56.1 - 1.713 (1.774 - 1.713)
Space group	P 43 21 2
Unit cell	79.3359 79.3359 37.7952 90 90 90
Total reflections	408044 (3026)
Unique reflections	12512 (576)
Multiplicity	32.6 (5.3)
Completeness (%)	92.76 (44.07)
Mean I/sigma(I)	33.70 (6.46)
Wilson B-factor	17.47
R-merge	0.1051 (0.1728)
R-meas	0.1063 (0.1927)
R-pim	0.0158 (0.0801)
CC1/2	0.998 (0.962)
CC*	0.999 (0.990)

Supplementary Table 3: **Merging statistics for the hen egg white lysozyme dataset processed with DIALS integration and scaling and merging with Aimless.** Statistics generated with `phenix.table_one`.

	I/σ		Multiplicity	
Time Point	Dark	2ms	Dark	2ms
Resolution Range				
19.31-3.59	55.74	55.84	2.67	2.67
3.59-3.02	48.35	48.63	3.36	3.38
3.02-2.72	42.35	42.21	3.62	3.63
2.72-2.53	32.32	32.30	3.85	3.82
2.53-2.40	29.94	29.22	4.34	4.35
2.40-2.29	30.52	30.58	4.48	4.51
2.29-2.20	25.37	24.77	4.32	4.32
2.20-2.13	26.25	25.75	4.81	4.79
2.13-2.07	20.99	20.08	4.66	4.67
2.07-2.02	19.68	18.73	4.99	5.01
2.02-1.97	18.01	17.15	4.77	4.76
1.97-1.93	16.51	15.72	5.34	5.32
1.93-1.88	15.66	14.65	4.45	4.43
1.88-1.84	13.68	12.84	4.78	4.76
1.84-1.80	11.42	10.46	4.24	4.25
1.80-1.76	10.99	10.17	4.12	4.09
1.76-1.73	9.78	8.96	4.21	4.20
1.73-1.69	8.20	7.64	3.61	3.59
1.69-1.64	8.26	7.49	3.28	3.30
1.64-1.53	7.15	6.52	2.05	2.06
Overall	22.52	22.01	3.90	3.90

Supplementary Table 4: **Average signal to noise and reflection multiplicity by resolution bin for the photoactive yellow protein dataset presented in Figure 4.**

Bin	Resolution Range	Completeness	CC_{int}	R_{int}	R_{split}	Scale	I/σ (Merged)	I/σ (Unmerged)
1	37.3478 - 4.8859	[2943/2953]	92.4%	22.5%	16.1%	0.963	8.389	48.658
2	4.8859 - 3.8780	[2947/2947]	94.5%	18.0%	12.8%	0.972	7.153	60.095
3	3.8780 - 3.3877	[2966/2966]	94.7%	19.0%	13.5%	0.953	6.377	49.758
4	3.3877 - 3.0780	[2934/2934]	93.7%	21.0%	14.9%	1.953	5.854	35.521
5	3.0780 - 2.8573	[2951/2953]	92.5%	23.1%	16.5%	0.954	5.598	28.723
6	2.8573 - 2.6888	[2940/2943]	90.6%	27.0%	19.5%	0.944	5.044	24.173
7	2.6888 - 2.5542	[2944/2949]	87.8%	29.5%	21.2%	0.941	4.825	20.027
8	2.5542 - 2.4430	[2932/2937]	86.0%	31.3%	22.6%	0.919	4.626	17.193
9	2.4430 - 2.3489	[2950/2957]	87.9%	29.7%	21.5%	0.936	4.532	16.009
10	2.3489 - 2.2679	[2934/2936]	85.2%	31.1%	22.4%	0.929	4.506	14.160
11	2.2679 - 2.1969	[2936/2950]	83.1%	34.5%	25.1%	0.916	4.107	12.133
12	2.1969 - 2.1341	[2919/2955]	77.8%	39.4%	29.1%	0.879	3.563	10.510
13	2.1341 - 2.0779	[2868/2926]	72.5%	42.8%	31.8%	0.867	3.246	9.415
14	2.0779 - 2.0272	[2747/2940]	70.7%	45.4%	34.0%	0.827	2.790	8.511
15	2.0272 - 1.9812	[2436/2929]	60.1%	54.0%	41.6%	0.779	2.278	7.496
16	1.9812 - 1.9390	[2162/2979]	49.0%	56.9%	45.3%	0.753	1.995	6.660
17	1.9390 - 1.9002	[1795/2933]	47.5%	60.6%	48.0%	0.719	1.730	6.052
18	1.9002 - 1.8643	[1331/2931]	49.4%	59.6%	49.1%	0.732	1.467	5.297
19	1.8643 - 1.8310	[922/2974]	29.4%	65.6%	53.6%	0.652	1.245	4.686
20	1.8310 - 1.8000	[642/2956]	36.3%	66.5%	56.1%	0.630	1.173	4.599
All	37.3478 - 1.8000	[50199/58948]	96.0%	24.6%	17.5%	0.933	4.101	—

Supplementary Table 5: **Thermolysin merging statistices reported by** `cctbx.xfel.merge`**.** Statistics are calculated by comparing even and odd images. Scale is the relative scale of even and odd results.

Supplementary References

[1] A. J. C. Wilson. The probability distribution of x-ray intensities. *Acta Crystallographica*, 2(5):318–321, 1949.

[2] Kevin M. Dalton, Jack B. Greisman, and Doeke R. Hekstra. rs-station/careless, November 2020. https://github.com/rs-station/careless.

[3] T. C. Terwilliger, P. D. Adams, R. J. Read, A. J. McCoy, N. W. Moriarty, R. W. Grosse-Kunstleve, P. V. Afonine, P. H. Zwart, and L.-W. Hung. Decision-making in structure solution using Bayesian estimates of map quality: the PHENIX AutoSol wizard. *Acta Crystallographica Section D: Biological Crystallography*, 65(6):582–601, June 2009.
