# Supplementary material for: A unifying Bayesian framework for merging X-ray diffraction data
Source: Nat Commun. 2022 Dec 15;13:7764. doi: 10.1038/s41467-022-35280-8 (PMC9755530; doi:10.1038/s41467-022-35280-8)
Supplement: Supplementary file 3 — Reporting Summary [file 41467_2022_35280_MOESM3_ESM.pdf]

nature portfolio

Corresponding author(s): Kevin M. Dalton, Doeke R. Hekstra

Last updated by author(s): Oct 22, 2022

Reporting Summary

Nature Portfolio wishes to improve the reproducibility of the work that we publish. This form provides structure for consistency and transparency in reporting. For further information on Nature Portfolio policies, see our Editorial Policies and the Editorial Policy Checklist.

Statistics

For all statistical analyses, confirm that the following items are present in the figure legend, table legend, main text, or Methods section.

n/a	Confirmed	
☐	☒	The exact sample size (n) for each experimental group/condition, given as a discrete number and unit of measurement
☒	☐	A statement on whether measurements were taken from distinct samples or whether the same sample was measured repeatedly
☐	☒	The statistical test(s) used AND whether they are one- or two-sided<br>*Only common tests should be described solely by name; describe more complex techniques in the Methods section.*
☒	☐	A description of all covariates tested
☐	☒	A description of any assumptions or corrections, such as tests of normality and adjustment for multiple comparisons
☐	☒	A full description of the statistical parameters including central tendency (e.g. means) or other basic estimates (e.g. regression coefficient) AND variation (e.g. standard deviation) or associated estimates of uncertainty (e.g. confidence intervals)
☒	☐	For null hypothesis testing, the test statistic (e.g. F, t, r) with confidence intervals, effect sizes, degrees of freedom and P value noted<br>*Give P values as exact values whenever suitable.*
☐	☒	For Bayesian analysis, information on the choice of priors and Markov chain Monte Carlo settings
☒	☐	For hierarchical and complex designs, identification of the appropriate level for tests and full reporting of outcomes
☐	☒	Estimates of effect sizes (e.g. Cohen's d, Pearson's r), indicating how they were calculated

Our web collection on statistics for biologists contains articles on many of the points above.

Software and code

Policy information about availability of computer code

Data collection	Collection of all diffraction data analyzed in this paper was described in references 9, 10, and 11 of the Online Methods, for hen egg white lysozyme (HEWL), photoactive yellow protein (PYP), and thermolysin, respectively. [***** RENUMBER REFERENCES ******]
Data analysis	Analysis was performed using Careless version 0.2.0 (0.2.3 for the "transfer learning" and "image layer" examples of the S-SAD dataset). DIALS version 3.1.4 was used to index and integrate observed reflections for hen egg white lysozyme. Aimless version 0.7.4 was used to merge the integrated intensities for hen egg white lysozyme data. We used XDS as updated on January 10, 2022. Precognition version 5.2.2 was used to index and integrate the polychromatic PYP data. cctbx.xfel version 2021.11.dev3+4.g05389c3054 was used to index, integrate, scale, and merge XFEL data for thermolysin. Refinement was performed in PHENIX version 1.18.2.<br><br>The source code and intermediate analysis used to generate all figures and tables is freely available from Zenodo (https://doi.org/10.5281/zenodo.6408750). The algorithm described herein is implemented in a python package which is available from our GitHub page (https://github.com/Hekstra-Lab/careless). It can be installed on Mac OS or Linux with the popular python package manager, pip.

For manuscripts utilizing custom algorithms or software that are central to the research but not yet described in published literature, software must be made available to editors and reviewers. We strongly encourage code deposition in a community repository (e.g. GitHub). See the Nature Portfolio guidelines for submitting code & software for further information.

Data

Policy information about availability of data

All manuscripts must include a data availability statement. This statement should provide the following information, where applicable:

- Accession codes, unique identifiers, or web links for publicly available datasets
- A description of any restrictions on data availability
- For clinical datasets or third party data, please ensure that the statement adheres to our policy

The refined lysozyme structure is deposited in the Protein Data Bank (PDBID: 7L84), and the raw lysozyme diffraction images are available through the SBGrid Data Bank (ID: 816). The intermediate analysis files used to generate all figures and tables is freely available from Zenodo (https://doi.org/10.5281/zenodo.6408750). Thermolysin XFEL data were obtained from CXI-DB 81. Unmerged intensities for each example are available from https://github.com/Hekstra-Lab/careless-examples. PDBID 2PHY was used to phase the difference electron density map in Figure 4, and PDBID 3UME was used as an excited state model for comparison in Figure 4. PDBID 2TLI [****** CORRECT? ANYTHING ELSE? *******] was used as a starting model for refinement in Figure 5.

Field-specific reporting

Please select the one below that is the best fit for your research. If you are not sure, read the appropriate sections before making your selection.

[x] Life sciences [ ] Behavioural & social sciences [ ] Ecological, evolutionary & environmental sciences

For a reference copy of the document with all sections, see nature.com/documents/nr-reporting-summary-flat.pdf

Life sciences study design

All studies must disclose on these points even when the disclosure is negative.

Sample size	Each set of "samples" reflects a single crystallographic experiment and was used as such. No additional effort was made to predetermine a suitable sample set size.
Data exclusions	No data were excluded for the examples in Figures 1-4. For Figure 5, one run from a larger XFEL dataset was arbitrarily chosen without prior assessment of the performance of the algorithm.
Replication	Careless generally supports the calculation of cross-validation measures with repeated random splitting of the data into two halves. Replication, e.g. in Figures 2 and 3, is for illustration purposes. The results are robust under cross validation.
Randomization	Careless relies on a standard random number generator that can (optionally) be seeded by the user. The kernels of the multilayer perceptron are initialized to the identity matrix and biases initialized as 0.
Blinding	Blinding was not relevant to the performed studies as we perform comparative analysis using default settings for each software.

Reporting for specific materials, systems and methods

We require information from authors about some types of materials, experimental systems and methods used in many studies. Here, indicate whether each material, system or method listed is relevant to your study. If you are not sure if a list item applies to your research, read the appropriate section before selecting a response.

Materials & experimental systems

n/a	Involved in the study	
[x]	[ ]	Antibodies
[x]	[ ]	Eukaryotic cell lines
[x]	[ ]	Palaeontology and archaeology
[x]	[ ]	Animals and other organisms
[x]	[ ]	Human research participants
[x]	[ ]	Clinical data
[x]	[ ]	Dual use research of concern

Methods

n/a	Involved in the study	
[x]	[ ]	ChIP-seq
[x]	[ ]	Flow cytometry
[x]	[ ]	MRI-based neuroimaging
